# miR-17 ~ 92 suppresses proliferation and invasion of cervical cancer cells by inhibiting cell cycle regulator Cdt2

**DOI:** 10.1007/s12672-023-00775-3

**Published:** 2023-09-14

**Authors:** Garima Singh, Sonika Kumari Sharma, Aastha Dorata, Samarendra Kumar Singh

**Affiliations:** Cell Cycle and Cancer Laboratory, School of Biotechnology, Institute of Science, Banaras Hindu University, Varanasi, UP 221005 India

**Keywords:** Cervical cancer, Cdt2, miR-17 ~ 92, Tumor suppressor, Invasion, Migration

## Abstract

Cervical cancer (CC) is the 4th most leading cause of death among women worldwide, and if diagnosed in late stages the treatment options are almost negligible. 99% of CC is caused by high-risk human papilloma viruses (HR-HPV). Upon integration into human genome, the encoded viral proteins mis-regulate various onco-suppressors and checkpoint factors including cell cycle regulators. One such protein is cell cycle S phase licensing factor, CDC-10 dependent transcript-2 (Cdt2) which has been reported to be highly upregulated in various cancers including CC. Also, in CC cells, several tumor suppressor miRNAs are suppressed, including miR-17 ~ 92 cluster. In this study, we report that miR-17 ~ 92 directly recruits to 3’UTR of Cdt2 and downregulates this oncogene which suppresses the proliferation, migration and invasion capabilities of the CC cell lines without affecting non-cancerous cells. We further show that suppression of Cdt2 by miR-17 ~ 92, blocks the cancerous cells in S phase and induces apoptosis, eventually leading to their death. Hence, our work for the first time, mechanistically shows how miR-17 ~ 92 could work as tumor suppressor in cervical cancer cells, opening up the potential of miR-17 ~ 92 to be used in developing therapy for cervical cancer treatment.

**Supplementary Information:**

The online version contains supplementary material available at 10.1007/s12672-023-00775-3.

## Introduction

Cervical cancer (CC) is one of the most lethal diseases caused by Human Papilloma Virus (HPV) with a survival rate of approximately 15% when diagnosed in late stages [1, 2]. It is the fourth most leading cause of death among women in the world, with approximately 604,000 new cases and 342,000 deaths reported annually, which is estimated to increase by 50% till 2030 [3]. HPV encoded oncoproteins E5, E6 and E7 interact with several cellular factors and drive them to mis-regulation, causing carcinogenic transformation of the healthy cells. The transformed cells have suppressed apoptotic checkpoint system including p53, pRb and several others resulting in hyperproliferation of cancerous cells [4, 5]. The E6 protein of HPV alone affects multiple genes to inhibit apoptotic signaling pathways and also activates telomerase reverse transcriptase to promote immortalization of cells. Apart from this, E6 also leads to ubiquitin mediated degradation of guardian of genome i.e., p53 protein via E6AP ubiquitin ligase system [6]. E6 stabilizes a major cell cycle regulator protein CDC-10 dependent transcript-2/Cdt2/DTL by recruiting a deubiquitinase, USP46, which was discovered to be essential for proliferation and survival of the cancer cells [7]. Cdt2 protein level has been reported to be highly up-regulated in various cancers, including cervical cancer [8].

Cdt2/DTL/DCAF2 is an essential component which was first discovered in fission yeast [9]. It is a substrate receptor DCAF (Ddb1-and Cul-4 associated factor) for CRL4 Cullin RING E3 ligase system, to form a complex CRL4^Cdt2^ which functions as a master regulator of cell cycle progression and genome stability [10]. CRL4^Cdt2^ E3 ubiquitin ligase system ensures the timely degradation of various cell cycle factors like p21, Set8 and Cdt1 etc., which are involved in DNA replication initiation, apoptotic checkpoint regulation and chromatin modification to prevent re-replication in S phase and is also required for the early G2/M checkpoint system [11, 12]. The levels of Cdt2 protein, like all other cell cycle regulators, is also maintained by several E3 ubiquitin ligase systems like CRL1^FBXO11^, CRL4^DDB2^, APC/C-Cdh1 etc. which leads to its elevated expression levels during G1 to S phase transition and decreased levels during mitosis [8].

Despite the availability of vaccines against HPV (16 and 18), due to the lack of awareness and proper planning in low and middle-income countries (accounting for approximately 90% of cases), there has been no significant decline either in cervical cancer cases or in mortality rates [13, 14]. The standard treatment available to cure cervical cancer is radiotherapy in combination with chemo and brachytherapy to which most of the patients do not respond and have high rate of relapse and lower survival rates. Therefore, we need a therapy which could be effective enough to majority of population. In this direction, miRNAs have emerged as target specific and effective therapeutic agents which could prove to be efficacious in regulating cancer progression [15, 16].

**Fig. 1:**
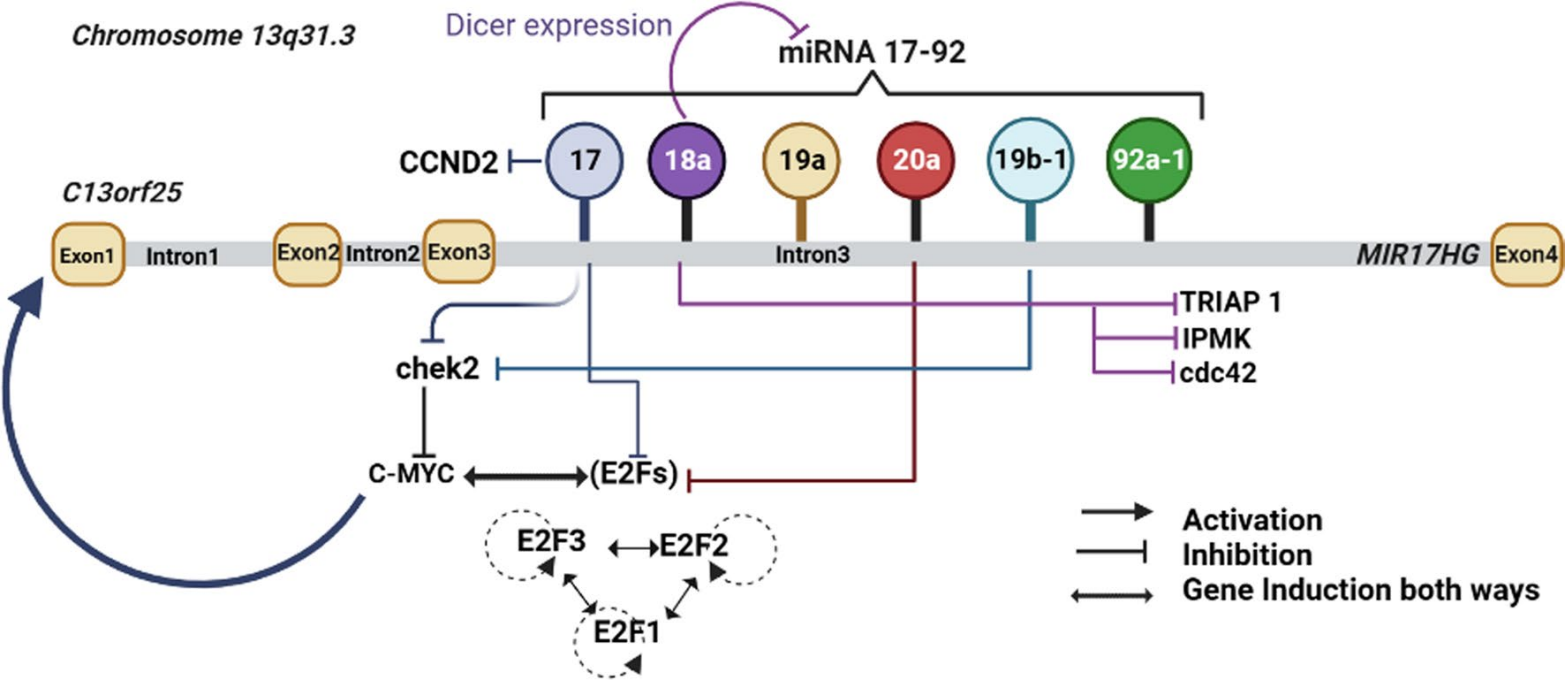
Coordinated transcriptional activation and the auto-regulatory feedback loop of miR-17 ~ 92. C-MYC activates the transcription of MIR17HG gene (coding the miR17-92 cluster). Upon increase in transcriptional activity of E2F or C-MYC, miR-17 ~ 92 inhibits the E2F/C-MYC translation. (Created in BioRender.com)

miRNAs are small non-coding RNAs (17–25 nucleotides in length) which play diverse roles in controlling gene expression by degradation/translation repression of target mRNAs. miRNA works by targeting 3'UTR (untranslated regions) sequences of mRNA which are complementary to their seed sequence of 6–8 base pairs [17]. Each miRNA can regulate several mRNAs (hence proteins) involved in regulating major processes like cell cycle, proliferation, apoptosis, development etc. Approximately, 60% of human genes are estimated to be regulated by miRNAs [18]. These miRNAs can either be encoded as a single miRNA (monocistronic) or as a cluster of multiple miRNAs (polycistronic). In the vertebrate genome, about 30% of miRNAs occur in the polycistronic cluster [19, 20]. miR-17 ~ 92 also known as oncomiR-1, is one of the polycistronic cluster, located in MIR17HG (miR-17 ~ 92 cluster host gene; non-protein coding) intron on chromosome 13 (13q31.3) [21–23] (Fig. 1). Expression of miR-17 ~ 92 is essential for development, regulation of cell cycle machinery, proliferation, and various other vital functions. Dysregulation of the same has been found in various cancers and diseases and is linked to their progression [24]. This cluster of miRNAs is regulated by direct interaction of two transcription factors, cellular myelocytomatosis oncogene (c-Myc) and n-Myc at the promoter region of miR-17 ~ 92 to initiate its transcription [25–27]. However, various studies have established both oncogenic and tumor suppressor roles of miRNA-17 ~ 92 cluster but the role of this cluster in cervical cancer remains unclear [21–23]. We became interested in miR-17 ~ 92 while looking for the effect of miR-34a on expression of Cdt2 protein [17]. In early screening we discovered that miR-17 ~ 92 cluster suppresses Cdt2 expression level in cervical cancer cell lines. We further explored the effect of miR-17 ~ 92 on proliferation, invasion and metastatic behavior of cervical cancer cells and discovered that it suppresses both proliferation and migration of these cells. This study is the first ever to report that miR-17 ~ 92 interacts with Cdt2 (at 3’UTR) and inhibits it at both transcript and protein level. Our findings, could pave the way to explore the possibility of using miR-17 ~ 92 towards developing it as miRNA therapy to manage cervical cancers in more specific manner.

## Materials and methods

### Cell lines and media

SiHa and HeLa cell lines (HPV positive cervical cancer cell lines) were purchased from the American Type Culture Collection (ATCC, USA). C33A (HPV negative cervical cancer cell line) and HEK293T (Human Embryonic Kidney cell line) were procured from National Centre for Cell Science (NCCS, Pune, India). The cells were cultured in Dulbecco’s Eagle Modified medium (DMEM, Gibco, USA) supplemented with 10% Fetal Bovine Serum (FBS, Gibco, USA) and 1% Pen-Strep (Penicillin–Streptomycin, Gibco, USA) unless mentioned otherwise and cultured at 37℃ temperature, 95% humidity and 5% CO_2_.

### Plasmids and miRNA

pcDNA 3.1 plasmid vector acquired from Addgene (Watertown, USA) was used as a control. miRNA-17 ~ 92 cloned in pcDNA 3.1 plasmid was obtained from Joshua Mendell lab [25]. Flag-Cdt2 plasmid was received as a gift from Dutta’s Lab (University of Virginia, USA). Rest of the miRNA mimics were purchased from Dharmacon (USA).

### Cell transfection

24 h before transfection, 0.1X10^6^ cancerous (HeLa, SiHa, C33A) and non-cancerous cells (HEK293T) were seeded in 6 well plates. The cells were then transfected with either miRNA mimics (10 nM) or plasmid vectors (pcDNA 3.1(2 µg), miR-17 ~ 92 (4 µg), Flag-Cdt2 (2 µg)) with turbofect™ (Thermo Fisher Scientific, Massachusetts, USA) according to the manufacturer’s protocol. The cells were then cultured for 48 h (as mentioned above).

### Cell proliferation assay

After transfection of cells (with miR-17 ~ 92 and respective control plasmids), they were harvested at consecutive days (for 4 days). The harvested cells were then counted in triplicates, using trypan blue exclusion method (0.4% trypan blue solution) with the help of haemocytometer, as well as by cell counter (TC-20, Biorad, California, USA). The growth curve was then plotted and the significance of treatment was calculated using unpaired student’s t-test.

### Western blot analysis

The cells were harvested and lysed using radioimmunoprecipitation assay (RIPA) buffer supplemented with PMSF (1 mM) and protease inhibitor cocktail (Cell Signaling Technology, Massachusetts, USA). The protein concentration was determined using Bradford protein assay (Sigma, Missouri, USA). The total protein was then resolved on SDS-PAGE (8–12%) and transferred onto PVDF membrane (Millipore, Massachusetts, USA). After the transfer, using protein ladder as reference, membranes were cut according to the protein of interest (as shown in the results). The further western blot protocol was followed as standard procedure [17] and** e**nhanced chemiluminescence (ECL) substrate (Bio-Rad, California, USA) was used to develop the blots. Documentation of the blots was done using chemidoc system (Azure Biosystems 600, California, USA).

### Cell invasion assay

After 48 h of transfection, the cells (0.1X10^6^) were washed with PBS and resuspended in serum-free DMEM. Cells were then added onto the precoated upper chamber (with 2 mg/ml ECM gel; Sigma-Aldrich, Missouri, USA) of transwell plate (Corning, New York, USA), and in lower chamber DMEM supplemented with 10% FBS was added. Cells were allowed to invade for 48 h in CO_2_ incubator. Cells that remained in the upper chamber were removed whereas the cells which invaded through the membrane were first fixed with 5% glutaraldehyde and then stained with 0.2% crystal violet solution in 2% ethanol. The cells were then counted under the phase contrast inverted microscope for quantification.

### Migration assay

Post 48 h of transfection, the cells (0.1X10^6^) were washed with PBS and resuspended in DMEM supplemented with 0.5% of FBS. Cells were then inoculated to the upper chamber of the transwell plate while in lower chamber DMEM supplemented with 0.5% FBS and 40 µg/ml collagen I (Sigma-Aldrich, Missouri, USA) was added. After incubation for 24 h in CO_2_ incubator, the migrated cells were fixed with 5% glutaraldehyde for 20 min and then stained with 0.2% crystal violet solution in 2% ethanol for 20 min. The cells were counted under phase contrast inverted microscope.

### Real time-qPCR

Both transfected and their control cells were harvested after 48 h of transfection followed by total RNA isolation using TRIZIN reagent (GCC Biotech, India). One step RT-qPCR kit (Invitrogen, Thermo Fisher Scientific, USA) was used according to manufacturer’s instructions to check the expression of Cdt2 at mRNA level by using the respective primers (forward and reverse [7]) as mentioned in the Table 1.

**Table 1:** Sequences of primers

Oligos/Primer	Sequences
qPCR Actin-F	TGAAGGCTTTTGGTCTCCCTG
qPCR Actin-R	TCAACTGGTCTCAAGTCAGTGT
qPCR Cdt2-F	CTTACAGTGGCGGGAGTTGG
qPCR Cdt2-R	GCATCAGGGTCGGAGGAAAA

### Flow cytometry

After transfection, the cells were trypsinized and washed with PBS. The cells were then fixed in 70% ethanol at − 20 °C overnight. Excess ethanol was washed with PBS followed by resuspension of the cells in 1 ml of staining solution (100 µg/ml propidium iodide, 100 µg/ml RNase-A in PBS and 0.1% Triton X-100) [28]. The stained cells were analyzed using the flow cytometer (Beckman Coulter, California, USA) and the results were then compared and analyzed using the CytExpert software provided with the flow cytometer system.

### Statistical analysis

All the western experiments were performed in biological triplicates. The Flowcytometry, growth curve, migration and invasion assays were performed in experimental triplicates. All the data is presented as either mean ± SD or mean ± SE and an unpaired student’s t-test was performed to calculate the significant value. Image J software was used to quantify the intensity of protein bands wherever applicable.

## Results

### miR-17 ~ 92 suppresses Cdt2 level in cervical cancer cells

**Fig. 2:**
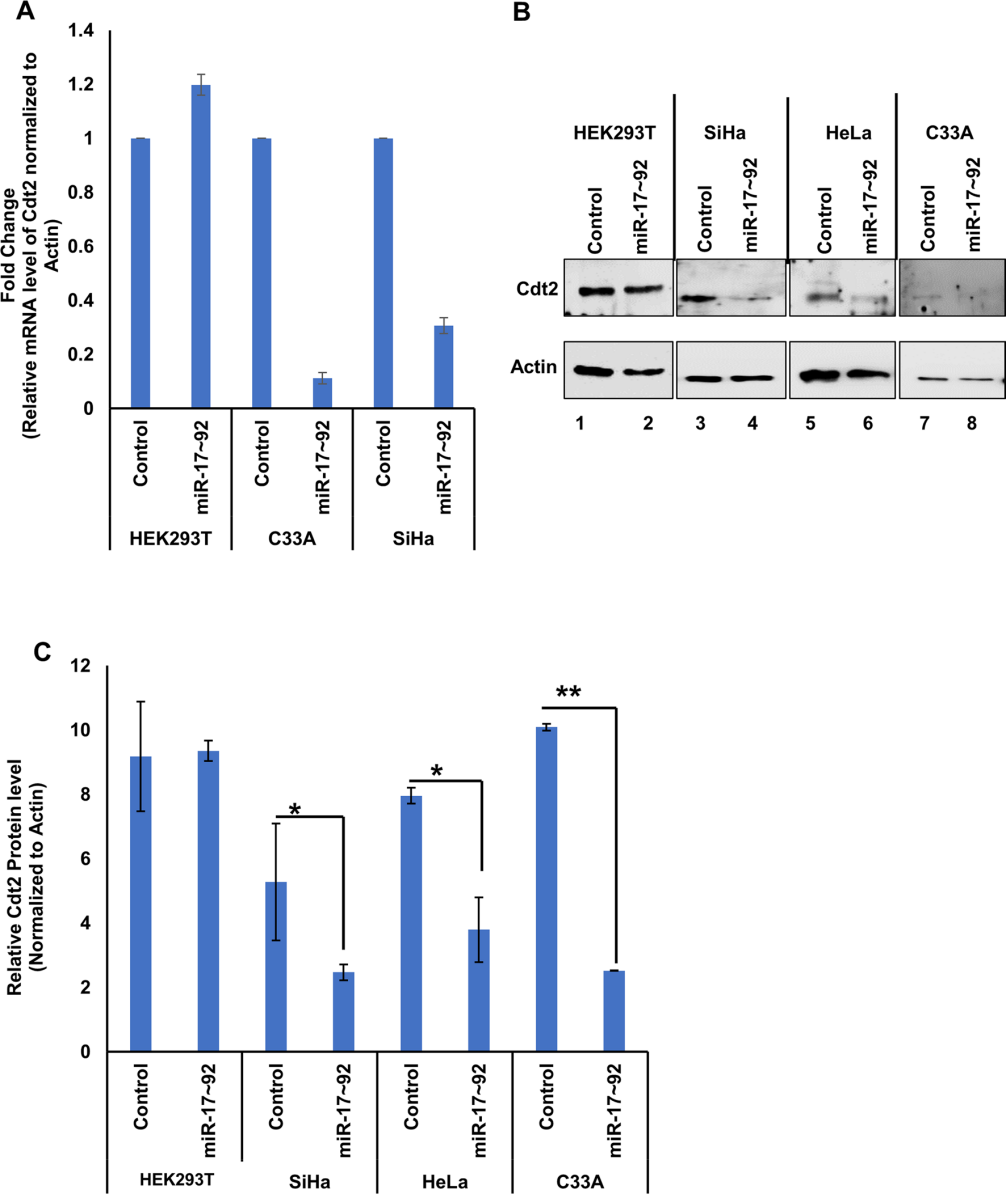
miR-17 ~ 92 over expression decreases Cdt2 expression in cervical cancer cells. **A** RT-qPCR analysis of mRNA level of Cdt2, 48 h after transfection of miR-17 ~ 92 in HEK293T, C33A and SiHa cell lines **B** Western blot for analysis of Cdt2 protein expression level, 48 h after transfection of miR-17 ~ 92 in HEK293T, SiHa, HeLa and C33A cell lines. **C** Quantification of Cdt2 protein level, 48 h after miR-17 ~ 92 transfection in HEK293T, SiHa, HeLa and C33A cell lines compared to vector control. The experiments were done in triplicates. Error bar depicts standard error (S.E.). *p value < 0.05 and **p value < 0.001

Previous studies have shown that in cervical cancer cells, level of Cdt2 is highly upregulated [7, 8]. In an early screen we have found that miR-17 ~ 92 suppresses Cdt2 protein levels. In order to check the complementarity between miR-17 ~ 92 cluster microRNA and Cdt2/DTL transcript we performed a microRNA databases search and found that according to miRDB, Micro T (Diana tool), miRWalk and RNA 22, miR-17 ~ 92 cluster interacts with Cdt2/DTL transcript at 3ʹUTR (Figure S1). To confirm the effect of miR-17 ~ 92 cluster on Cdt2/DTL transcript in cervical cancer cells, we transfected the miR-17 ~ 92 expressing plasmid in HPV positive cervical cancer cell; SiHa, HPV negative cervical cancer cell; C33A and non-cancerous cell line; HEK293T. We observed that there is a significant decrease in the transcript levels of Cdt2 both in HPV positive (SiHa) and negative cervical cancer (C33A) cell lines, but not much effect in non-cancerous (HEK293T) cell line (Fig. 2A). To check that if this effect is getting translated at protein level, we performed western blot analysis for HPV positive and negative cervical cancer cells to be confirmed that there is a significant amount of decrease in the levels of Cdt2 protein in cancerous cells (Fig. 2B, Lanes 3–8 and Fig. 2C), while no such effect could be observed in non-cancerous cells (Fig. 2B, Lanes 1 and 2 and Fig. 2C).

### miR-17 ~ 92 stabilizes Set8 and blocks cell cycle in the S phase of cervical cancer cells

**Fig. 3:**
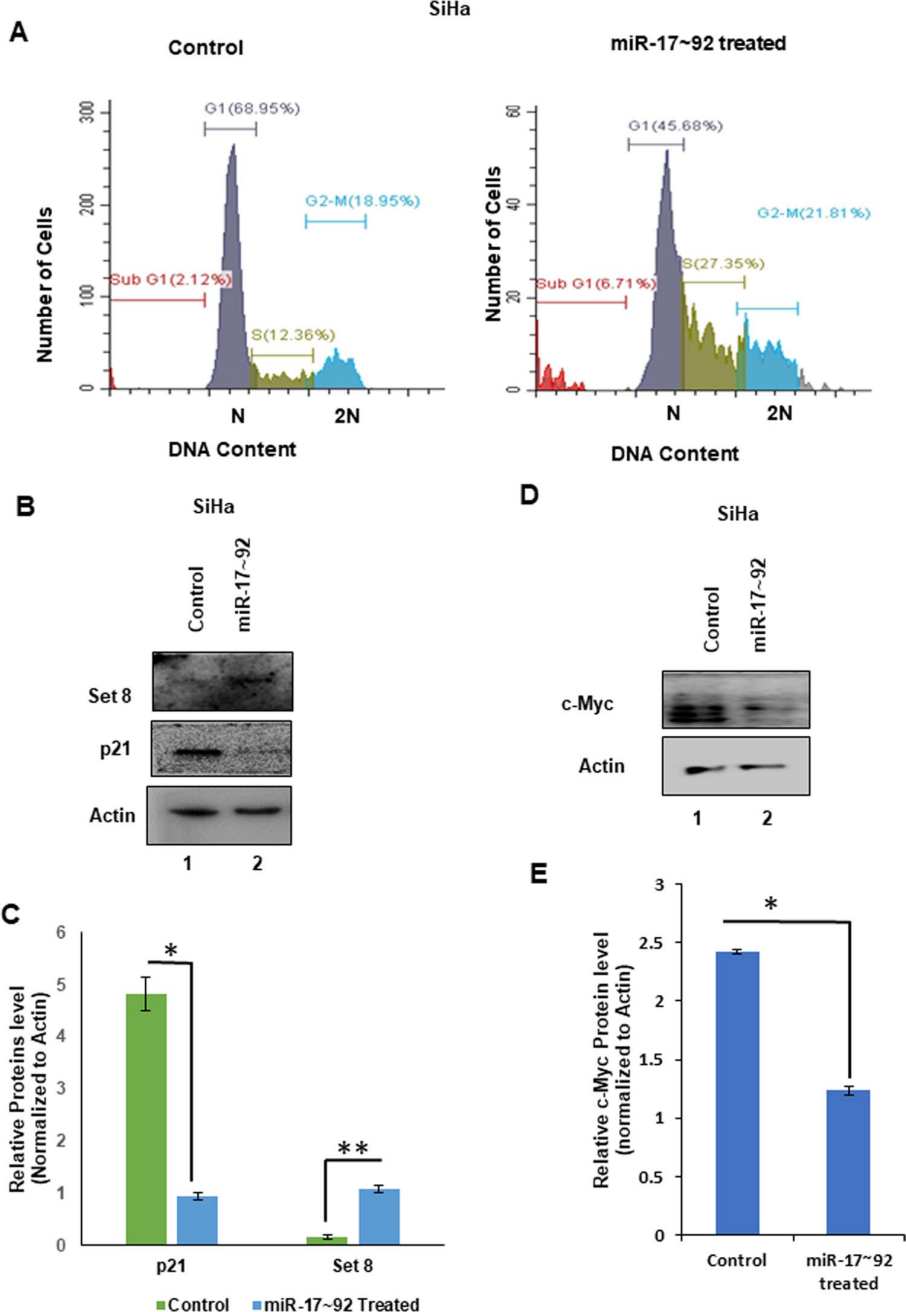
miR-17 ~ 92 ectopic expression causes S phase arrest in cervical cancer cells. **A** Flowcytometric analysis of SiHa cells post 48 h of transfection of miR-17 ~ 92. **B** Western blot for analysis of Set8 and p21 protein expression level 48 h after transfection of miR-17 ~ 92 in SiHa Cells. **C** Quantification Set8 and p21 protein level post 48 h of miR-17 ~ 92 transfection in SiHa Cells compared to vector control. **D** Western blot for analysis of c-Myc protein expression level 48 h after transfection of miR-17 ~ 92 in SiHa Cells. **E** Quantification c-Myc protein level 48 h post miR-17 ~ 92 transfection in SiHa Cells compared to vector control. The experiments were done in triplicates. Error bar depicts standard error (S.E.). *p value < 0.05 and **p value < 0.001

From previous research findings, it has been established that Cdt2 regulates the level of p21 and Set8 by proteasomal degradation [7, 10], hence, we next checked the level of these two proteins upon miR-17 ~ 92 ectopic expression in cervical cancer cells. We observed that decrease in Cdt2 levels, leads to significant stabilization of Set8 protein levels (Fig. 3B, C and S4), both in HeLa and SiHa cells. In contrast to Set8 expression, p21 expression is decreased upon miR-17 ~ 92 overexpression in both the above cell lines (Fig. 3B, C and S4).

Since Cdt2 and Set8 both are important for S phase progression of the cell cycle [10, 29]; therefore, we did flow cytometry analysis to see the effect of Cdt2 suppression upon miR-17 ~ 92 overexpression in these cancerous cells (SiHa, Fig. 3A, control Vs treated). We observed that cells are getting significantly arrested at S phase of the cell cycle, with an increase of cell population from 12.4 to 27.4% in control versus miR-17 ~ 92 treated cells respectively, which is more than twofold, upon miR-17 ~ 92 treatment. Also, we could observe moderate arrest of the cell cycle in G2/M phase with increases in the cell population from 18.9% to 21.8% (Fig. 3A, control vs treated) and also increase in sub G1 population which represents the apoptotic cells (Fig. 3A, control vs treated).

Since c-Myc and miR-17 ~ 92 cluster works in an auto-regulatory feed-back loop; therefore, next we wanted to confirm the effect of miR-17 ~ 92 on c-Myc. As reported in previous literatures, we too confirmed that ectopic expression of miR-17 ~ 92 cluster indeed significantly suppresses the level of c-Myc in cervical cancer cells (Fig. 3D and E).

### miR-17 ~ 92 suppresses the proliferation of cervical cancer cells

**Fig. 4:**
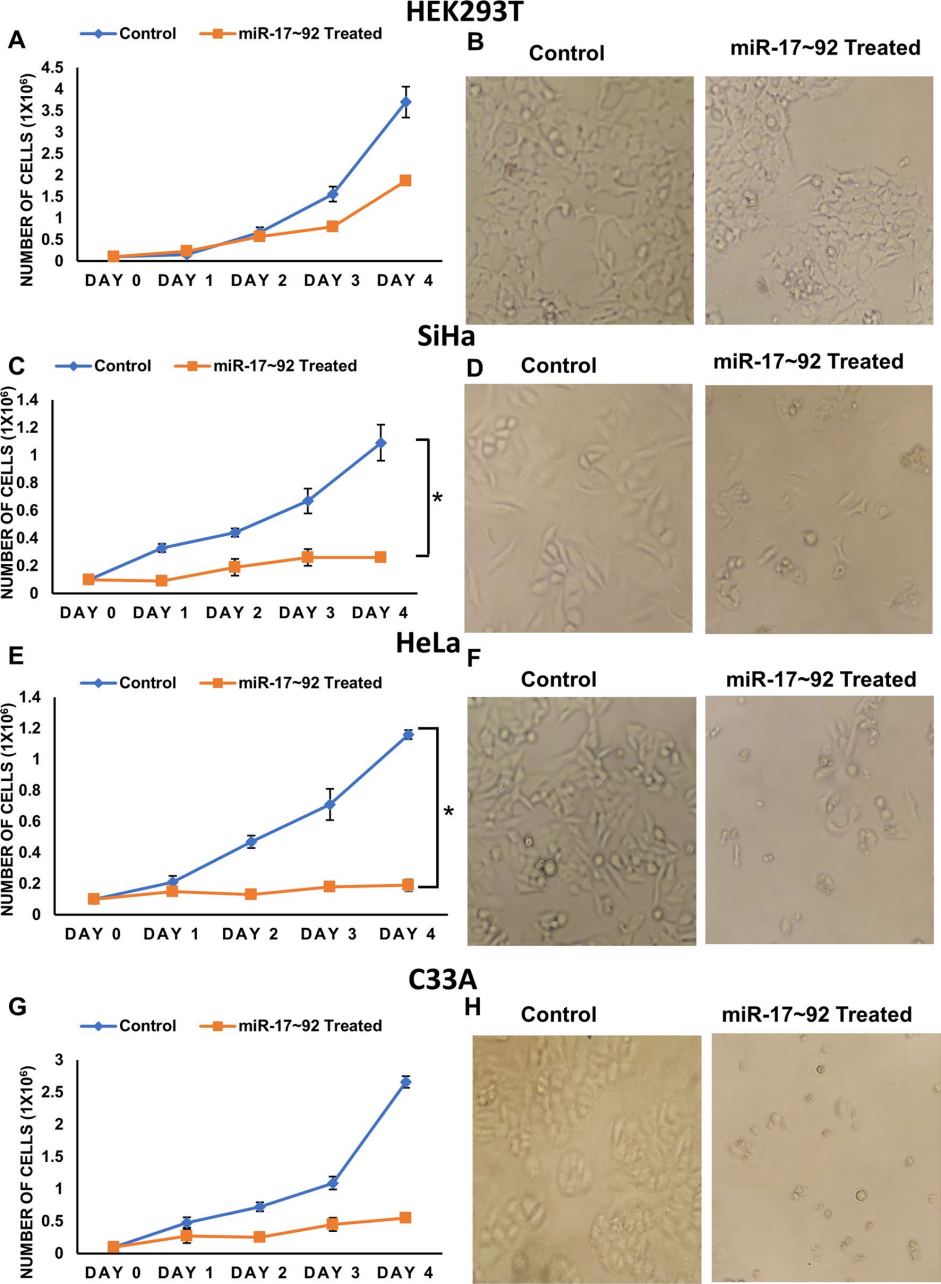
miR-17 ~ 92 was ectopically expressed and the proliferation was observed from day of transfection till day 4. **A** Growth curve of HEK293T cells with its respective control. **B** Control and treated HEK293T cells after 48 h of transfection under phase contrast microscopy. **C** Growth curve of SiHa cells along with its respective control **D** Control and treated SiHa cells under phase contrast microscopy after 48 h of transfection. **E** Growth curve of HeLa and its respective control. **F** Control and treated HeLa cells after 48 h of transfection under phase contrast microscopy. **G** Growth curve of C33A cells and its respective control. **H** Control and treated C33A cells after 48 h of transfection under phase contrast microscopy. The experiment was done in triplicate. Error bar represents S.D. *p value- < 0.05, **p value < 0.001

We have shown above that miR-17 ~ 92 suppresses Cdt2 level in cervical cancers cells and since Cdt2 is one of the major regulators of cell proliferation in cervical cancers [17, 30], we next wanted to check the effect of miR-17 ~ 92 overexpression on growth and proliferation of the same. We observed that miR-17 ~ 92 expression significantly inhibits the proliferation of HPV positive cervical cancer cells (both HeLa and SiHa, Fig. 4C–F) and also of HPV negative cervical cancer cells (C33A, Fig. 4G and H) while there is no such effect on non-cancerous HEK293T cells, where the growth decreased at 3^rd^ day has been compensated by day 4 (Fig. 4A, B and S2). Also, the phase contrast microscopy images show that, upon miR-17 ~ 92 transfection the cervical cancer cells have incurred morphology and have acquired more rounded structure in comparison to wild type cell lines (Fig. 4D, F and H and S3B-S3D) while no such change has been observed in the morphology of HEK293T cells upon transfection (Fig. 4B and S3A).

**Fig. 5:**
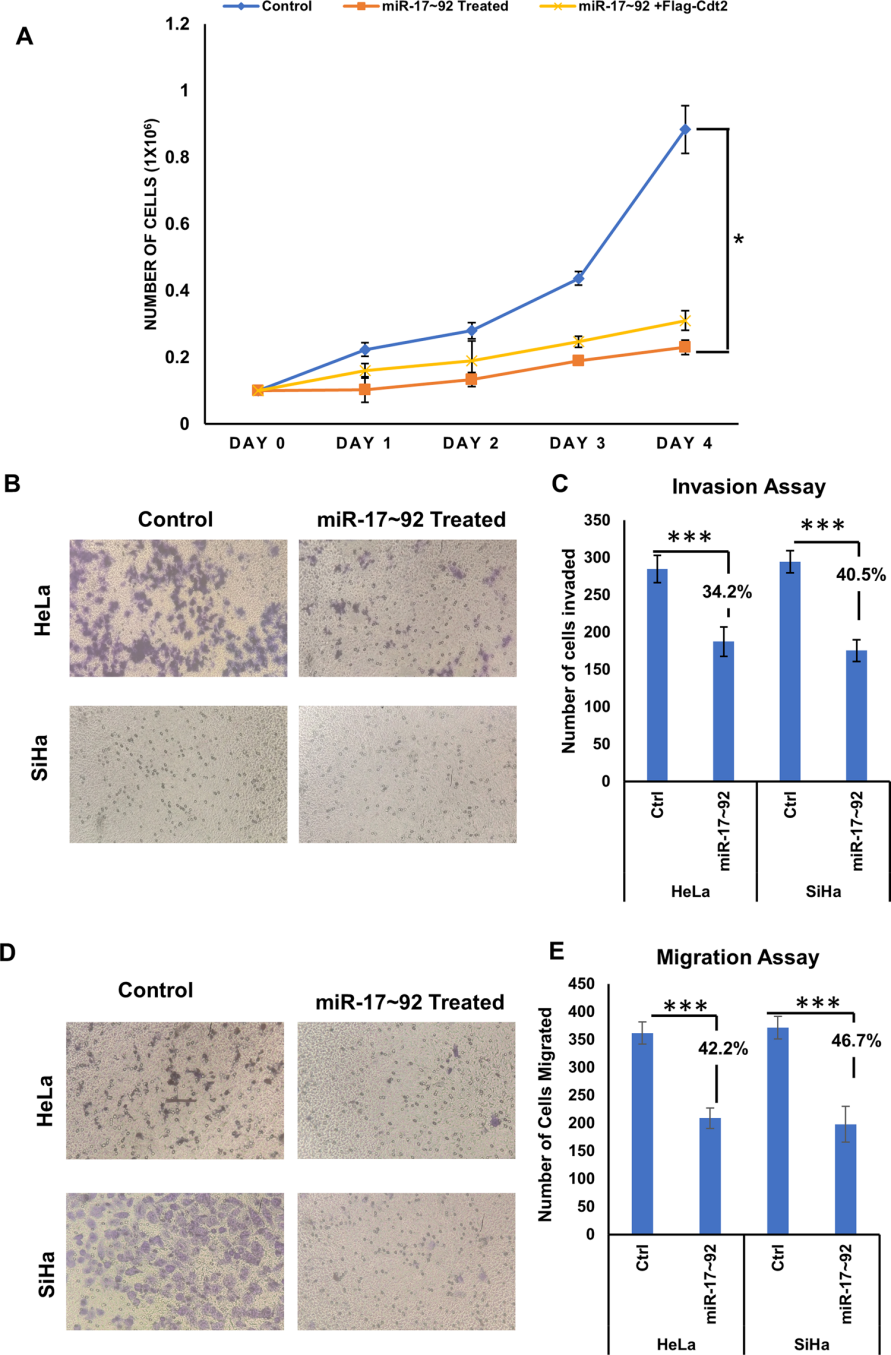
**A** miR-17 ~ 92 and Flag-Cdt2 was ectopically expressed individually/in cocktail and the proliferation was observed from day of transfection till day 4. The experiment was done in triplicate and error bar depicts standard deviation (S.D.). Effect of miR-17 ~ 92 ectopic expression on transwell migration and invasion of cervical cancer cells. **B** Phase contrast microscopy images of control and miR-17 ~ 92 treated SiHa and HeLa cells representing the change in invasion ability of treated cells against their respective controls. **C** Graphical representation of invasion assay of HeLa and SiHa cells along with their respective controls. **D** Images representing the migration of miR-17 ~ 92 treated HeLa and SiHa cells along with their respective controls. **E** Graphical representation of migration assay of HeLa and SiHa cells along with their respective control. 8 different areas were selected randomly and number of cells invaded and migrated were counted. The experiments were done in triplicates. Error bar depicts standard error (S.E.). *p value < 0.05 and ***p value < 0.0001

Further, we wanted to confirm if decreased proliferation rate in cervical cancer cells upon miR-17 ~ 92 expression is because of suppression of Cdt2. To ensure this, we transfected SiHa cells with either miR-17 ~ 92 expressing plasmid alone or co-transfected miR-17 ~ 92 plasmid in combination with Cdt2 expressing plasmid. The results showed that ectopic expression of Flag-Cdt2 along with miR-17 ~ 92, could rescue the suppressed growth of cervical cancer cells (Fig. 5A).

### Upregulation of miR-17 ~ 92 suppresses both cell migration and invasion of cervical cancer cell lines

After establishing that miR-17 ~ 92 expression suppresses proliferation of cervical cancer cells, we then checked its effect on the cell migration and invasion capabilities. The transwell invasion and migration assays show that, miR-17 ~ 92 reduces the invasion ability of HeLa cells by 34.2% and of SiHa cells by 40.5% in comparison to the control (Fig. 5B–C). We also observed suppression in the migration ability of HeLa cells by ~ 0.7 fold, and that of SiHa cells by ~ 0.9 fold (Fig. 5D and E). Hence, invasion and migration abilities of cervical cancer cells are significantly suppressed upon miR-17 ~ 92 overexpression.

## Discussion and conclusion

Cervical cancer is fourth most aggressive and fatal cancer among women worldwide associated with the mutation and deletion in miRNA genes and their aberrant expression [3, 31]. One such miRNA is miR-17 ~ 92 cluster (encoded by 13q31.3), displaying both tumor suppressor and oncogenic functions and works in an autoregulatory manner in order to maintain the growth and proliferation of the cells [21–23]. In cervical cancer cases specifically in HPV infected one, E6 protein apart from affecting other pathways also modulate the expression of miRNA-92 from the cluster and increase the proliferation of the infected cells [32]. However, nothing much is known about the mechanism behind regulation of cell cycle factors by miR-17 ~ 92 in cervical cancers.

In this study we show that over-expression of miR-17 ~ 92 suppresses the proliferation in cervical cancer cells while non-cancerous cells are not much affected. To confirm the tumor suppressor role of miR-17 ~ 92, we performed miRNA databases (miRDB, miRWalk etc.) search to identify the potential targets of miR-17 ~ 92 cluster and discovered that several miRNAs from cluster indeed suppresses this essential cell cycle factor Cdt2/DTL, which is involved in G1 to S phase transition [10, 33, 34] by targeting its 3’UTR (Figure S1). This *in-silico* information was validated by RT-qPCR, where we have confirmed that miR-17 ~ 92 indeed suppresses the mRNA level of Cdt2 both in HPV negative and positive cervical cancer cells but there was no suppression in non-cancerous cells (Fig. 2A). On the basis of complementarity of miRNA seed sequences to the 3’UTR of the target mRNA, miRNAs have the ability to either degrade the mRNA or inhibit the translation of protein [18]. Our results indicates that miR-17 ~ 92 might be a natural suppressor of Cdt2. Since Cdt2 levels are quite higher in cervical cancer cell lines, overexpression of miR-17 ~ 92 (which itself is suppressed in cervical cancer cells) bring back Cdt2 levels to normalcy, which in turn results in inhibition of proliferation of these cancerous cells. This suppressive role of miR-17 ~ 92 in the expression of Cdt2 was supported by the downregulation of Cdt2 protein only in cervical cancer cells (Fig. 2B Lane 3–8 and 2C) while not much significant change was observed in non-cancerous cells (Fig. 2B Lane 1–2 and 2C). This confirms that suppressive nature of miR-17 ~ 92 on Cdt2 is specific to cervical cancer cells only (where Cdt2 has been reported to be up-regulated), opening up avenue to explore miR-17 ~ 92 as a specific therapeutic candidate for cervical cancer.

Cdt2 is an adapter protein of CRL4 E3 ubiquitin ligase system which leads to the ubiquitin mediated degradation of cell cycle pro-apoptotic and onco-suppressor factors p21 and Set8 [10, 11, 35]. miR-17 ~ 92 mediated suppression of Cdt2, stabilizes its downstream target Set8 protein (Fig. 3B, C and S4), which is a major cell cycle regulator that licenses DNA replication and promotes efficient DNA repair [7]. It also plays a major role in regulating expression of genes critical for S phase progression. Further, it has been established in literature that degradation of Set8 is essential for S to M phase transition, hence, increased Set8 level upon ectopic expression of miR-17 ~ 92 in cervical cancer cells (SiHa), causes extensive S phase arrest [29] and the cells could not escape into G2/M. The moderate G2/M arrest [12] can be characterized by decrease in Cdt2 level upon miR-17 ~ 92 ectopic expression. In contrast to Set8 the level of p21 decreases upon miR-17 ~ 92 ectopic expression (Fig. 3B, C and S4) as p21 is also one of the targets of this cluster.

It has been already reported, that in cervical cancer, Cdt2 is upregulated and is responsible for the proliferation, re-replication and tumorigenesis [7], therefore suppression of Cdt2 by miR-17 ~ 92 shows significant reduction in growth and proliferation of HPV positive cervical cancer cells (Fig. 4C–F) which can be rescued by ectopic expression of Cdt2 protein (Fig. 5A). Also, in HPV negative cells (C33A) there is suppression in growth and proliferation, though the suppression is not significant enough (Fig. 4G–H). Whereas, in non-cancerous cells (HEK29T), Cdt2 level is not suppressed by miR-17 ~ 92 expressions, the growth and proliferation remain unaffected. The other reason for the inhibition of growth, in these cells, could be suppression of c-Myc level upon miR-17 ~ 92 expression (Fig. 3D and E). miR-17 ~ 92 works in an auto-regulatory loop with c-Myc, where miR-17 ~ 92 suppresses the overly expressed c-Myc, turning off its oncogenic signaling [21, 22, 25] leading to suppression of growth in cancerous cells. Additionally, miR-17 ~ 92 overexpression inhibits the cell migration and invasion ability of cervical cancer cells (Fig. 5B–E) probably by inhibiting the proliferation of cells.

**Fig. 6:**
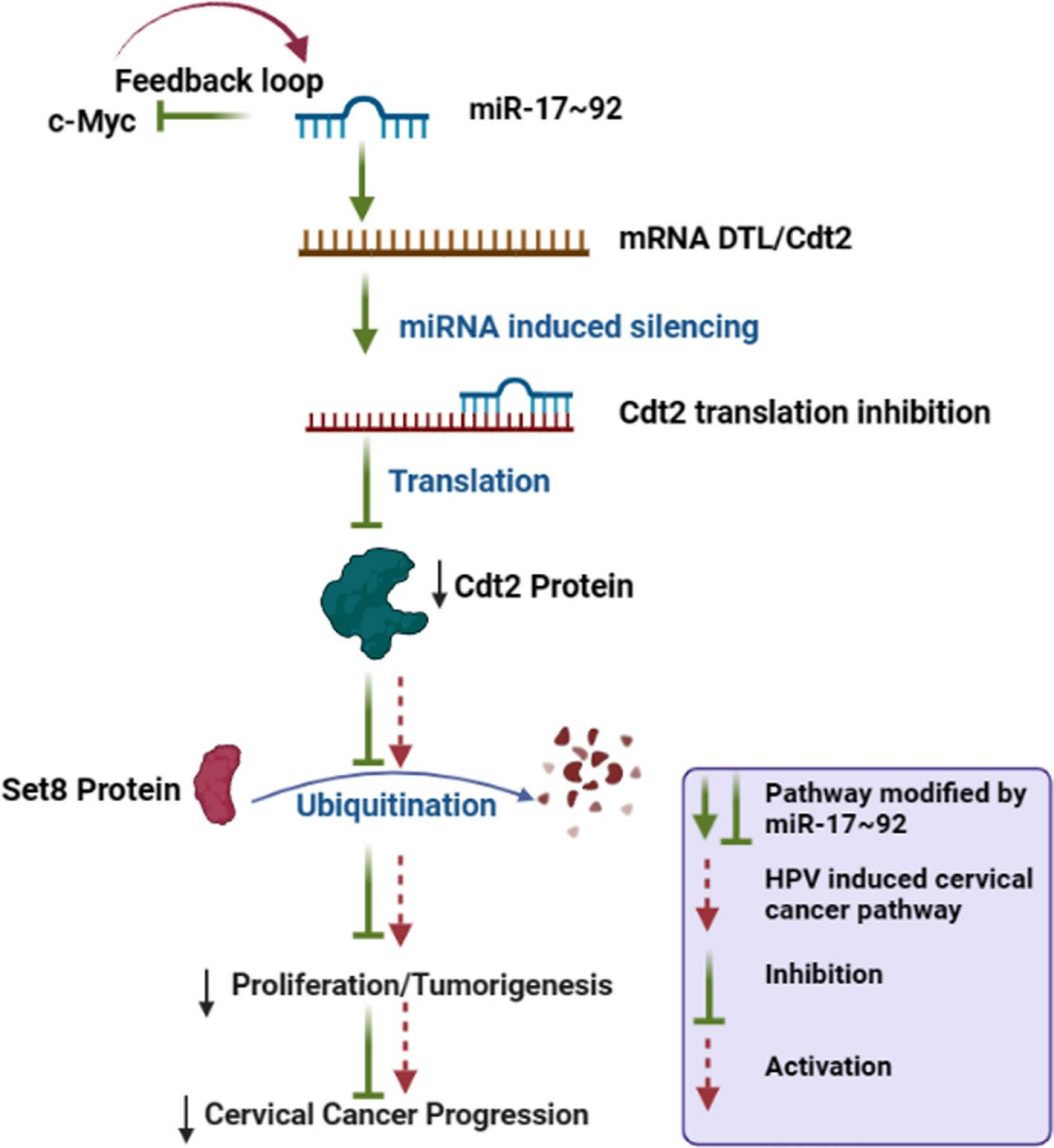
miR-17 ~ 92 ectopic expression suppresses Cdt2 at mRNA level, leading to stabilization of Set8 and decreases proliferation and tumorigenesis (Created in BioRender.com)

Hence, our study for the first time (ever to be reported) proposes that miR-17 ~ 92 cluster act as a tumor suppressor in cervical cancer cells by targeting Cdt2, one of the master regulators of cell cycle and re-replication. Our results also confirm, that miR-17 ~ 92 suppresses mRNA of Cdt2 via miRNA induced silencing, leading to inhibition of Cdt2 protein production. Suppression of Cdt2 protein eventually leads to stabilization of a replication licensing factor Set8 causing S phase arrest of the cancerous cells. This in turn leads to inhibition of proliferation, invasion and migration abilities of the cervical cancer cells (Fig. 6), while there is no such effect on the non-cancerous cells. Our study opens up possibilities to explore the potential of using miR-17 ~ 92 towards cervical cancer treatment, which could prove to be much cheaper and specific therapeutic option for cervical cancer.

### Supplementary Information

Additional file 1 (PDF 955 KB)

## Data Availability

All data generated or analyzed during the study are included in this article (and its supplementary information files) and also available from the corresponding author on reasonable request.
