# Supplementary figures and images for: miR-17 ~ 92 suppresses proliferation and invasion of cervical cancer cells by inhibiting cell cycle regulator Cdt2
Source: Discov Oncol. 2023 Sep 14;14:172. doi: 10.1007/s12672-023-00775-3 (PMC10501107; doi:10.1007/s12672-023-00775-3)

Figure S1

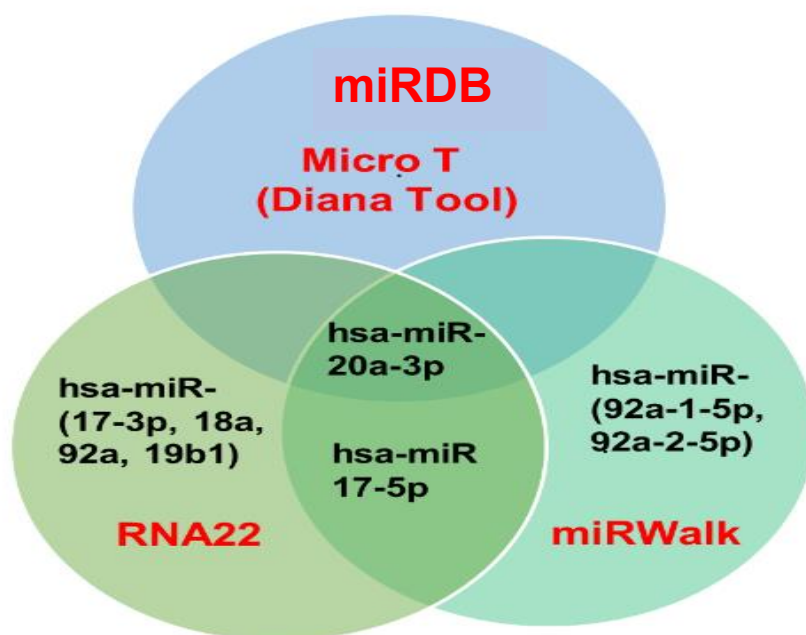

Figure S2

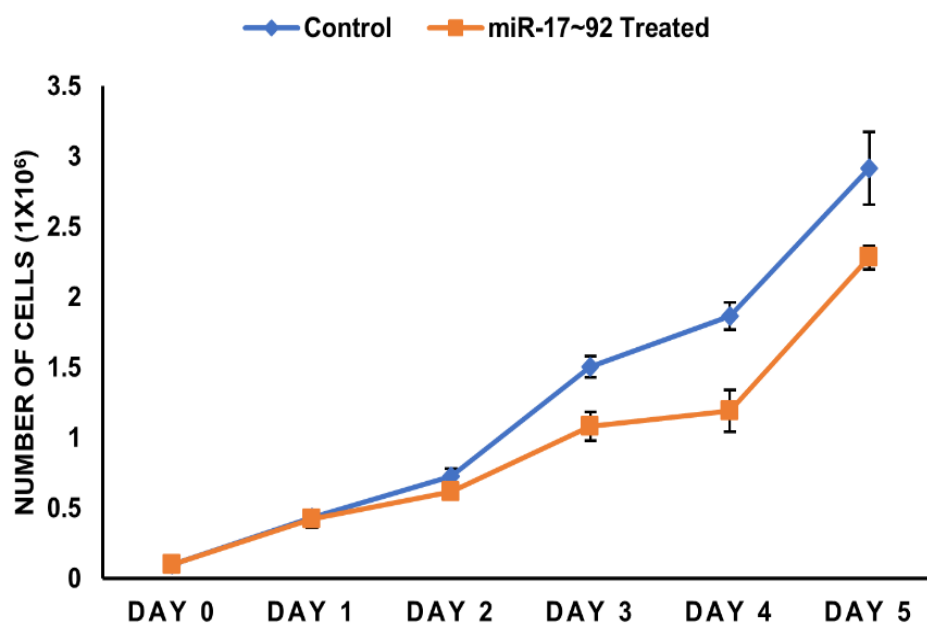

Figure S3

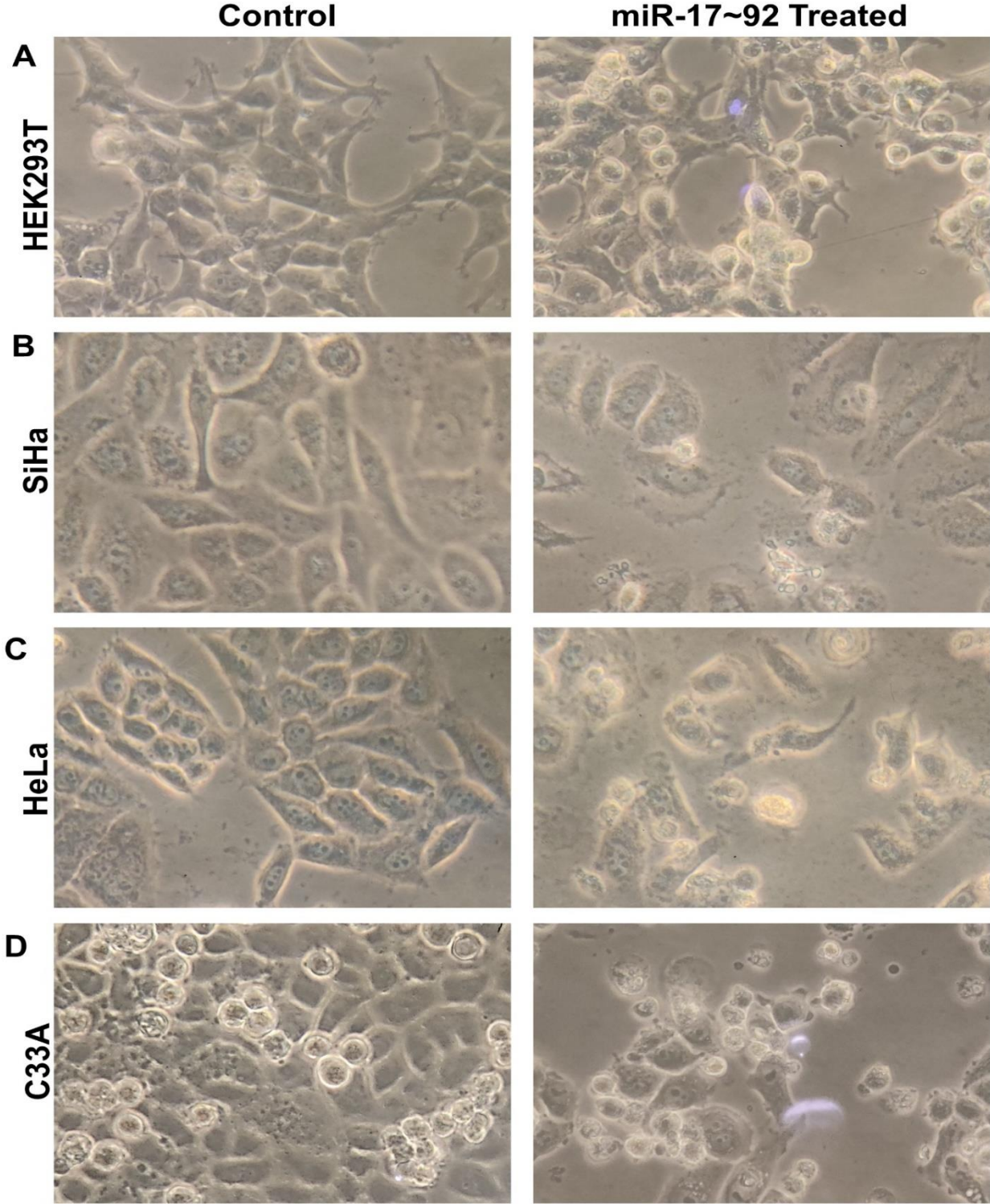

Figure S4

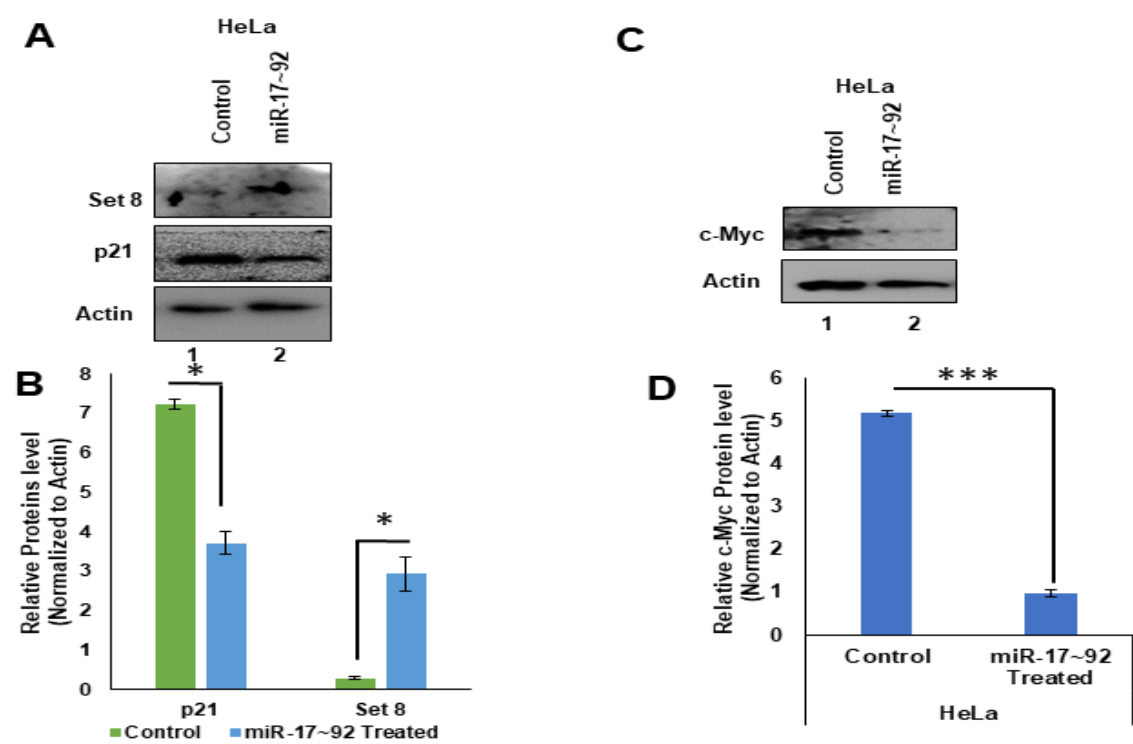

Supplement: Supplementary file 1 — Additional file 1 (PDF 955 KB) [file 12672_2023_775_MOESM1_ESM.pdf]
